# The King's Fund and the Hospitals

**Published:** 1904-12-24

**Authors:** 


### The Hospital.

A Journal of

The Medical Sciences and Hospital Administration.

Vol. XXXVII.—No. 952. DECEMBER 24, 1904.

### THE KING'S FUND AND THE HOSPITALS.

"We have shown our subscribers that we are endeavouring, as far as possible, to assist the hospitals to lay out our grants economically, and to husband their resources; that we are willing to help them, if they so desire, to settle any vexed question that may arise with regard to difficulties of administration, whether internal or external, or on matters concerning reconstruction, removal, or amalgamation." Thus spoke the Prince of Wales, as President of the King's Fund, at Marlborough House on the 19th inst. His whole speech, which will be found in another column, is a statesmanlike utterance, which will no doubt be studied and weighed by the managers of every voluntary hospital throughout the country. It is very suggestive, and proves the real grasp which the Prince has obtained of the most pressing hospital questions. The greater the knowledge of hospital questions, the more the speech will appeal to those who read it. The King's Hospital Fund has accomplished much, and this speech affords guarantees, that with the hearty co-operation of the hospital managers, it will, in time, promote great changes, in many directions, which cannot fail to increase the popularity and efficiency, as well as the resources, of these noble institutions.

The Prince points out with regret that the revenue of the Fund for the year 1904 showed no material advance, but the contribution of the League of Mercy had increased by forty per cent. He properly ascribes this result to the general depression, which it is gratifying to find had little apparent effect upon the humbler supporters of hospitals, who gave their shillings through the League of Mercy. Seeing that money is scanty, in these times of depression, the Prince rightly thinks, that a way may be found of increasing the means at the disposal of the hospitals for promoting their increasing usefulness by decreasing the expenditure, without impairing the extent or efficiency of the work the hospitals do. In support of this view he states, that the expenditure in 1903 of some of the sixteen largest general hospitals, which received more than one-half of the grants made last year, exceeded the average expenditure, upon items over which control exists, by £40,000, in round figures.

The Prince does not say that all this money can be saved at once, but he believes that when it is realised, that one hospital is doing the same work as another, equally well but at a lower cost, then economy will be enforced, efficiency promoted, and the resources of each such hospital must so be considerably increased. The Prince further emphasises his point by mentioning the following discrepancies shown by a comparison of the prices of the principal goods used in the different hospitals. Thus, there is a difference of no less than 3¼d. per lb. between the highest and lowest prices paid for the same description of beef, of 3½d. per lb. for legs of mutton, of 1s. 1d. in the price of fowls, of 2½d. per gallon in the price of milk, and of 7d. per lb. in the price of tea. In drugs and household articles there are similar discrepancies.

There may of course be reasons to account for some of these differences, but when everything is taken into account, the discrepancies shown, are so marked, as to call for a careful inquiry, on the part of each hospital. Already one of the largest and best managed hospitals has gratefully acknowledged the efforts of the King's Fund, and, as a result of the comparisons referred to by the Prince, various economies have been effected which have resulted in a considerable saving. The Prince further points out the advantage, so far as prices paid are concerned, of arranging for large rather than small contracts. He suggests the possibility of some of the smaller hospitals combining to make a large contract and thus gain the advantage enjoyed by the greater institutions. He offered the services of the King's Fund to organise the preliminary arrangements, and suggested a conference with a view to encourage hospital managers to meet and discuss these important questions.

Everybody has been favourably impressed by the important manifesto of the President of the King's Fund. King Edward may well be proud of the steady progress and success of the King's Fund under the inspiriting direction of the Prince of Wales. The majority of the London hospitals have always been behind the best of the provincial and Scotch hospitals in matters of administration. We have shown[1] that a great extra-metropolitan hospital with a large and prosperous medical school, when administered by an able medical superintendent, can so control its expenditure in every department, that a

[1] The Hospital, Dec. 10th, pp. 195–5.

practically uniform cost per bed can be maintained year by year. It is time that the London hospitals devoted all the energies of their managers to the enforcement of economy throughout every department. Guy's under an able medical superintendent has shown the way. The sooner a reform of system is made in other great London hospitals the sooner will the present faults be permanently redressed.

